# Supplementary material for: Development of Digitally Obtainable 10-Year Risk Scores for Depression and Anxiety in the General Population
Source: Front Psychiatry. 2021 Aug 13;12:689026. doi: 10.3389/fpsyt.2021.689026 (PMC8414584; doi:10.3389/fpsyt.2021.689026)
Supplement: Supplementary file 1 [file Data_Sheet_1.docx]

***Supplementary Material***


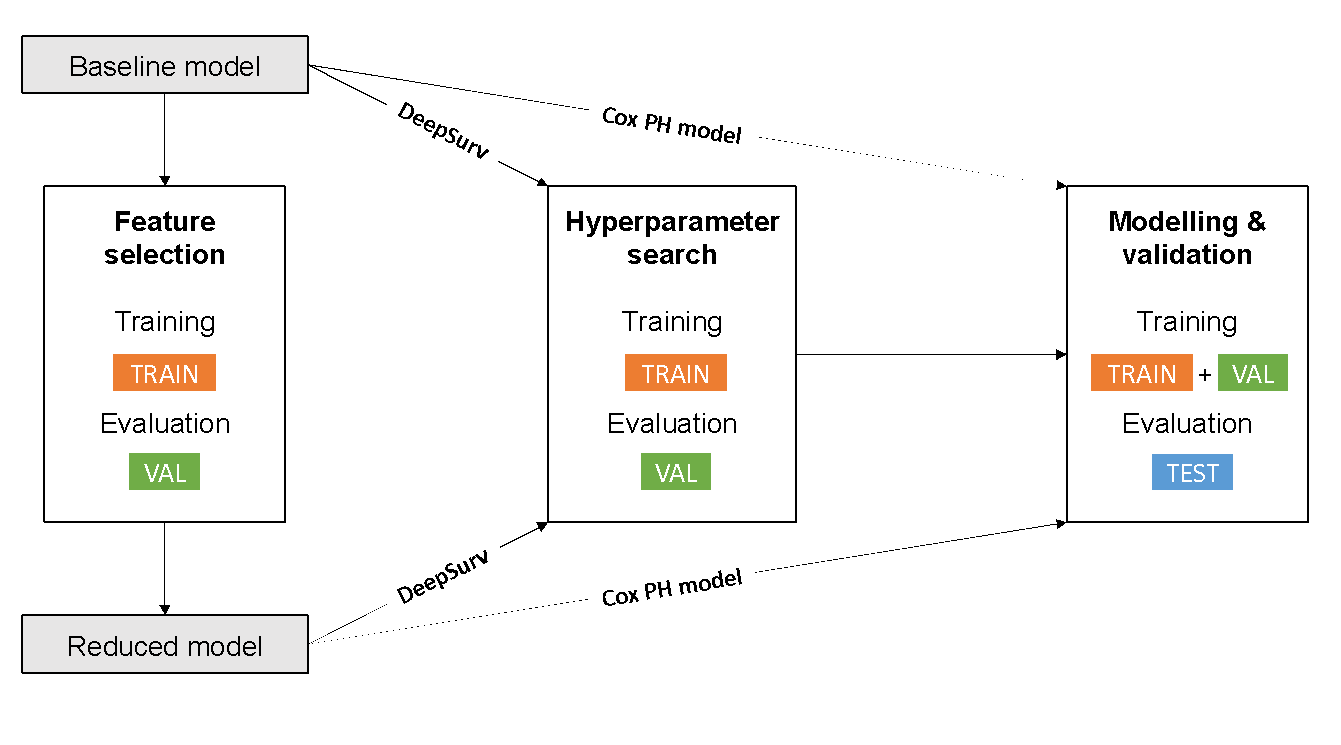


**Supplementary Figure 1: Diagram of the study design**. Dataset partitions used for each step of the analysis and modelling are shown in coloured rectangles. Final model was trained using merged train and validation dataset and was subsequently internally validated using the unseen test dataset. PH = proportional hazards, TEST = test dataset, TRAIN = train dataset, VAL = validation dataset.


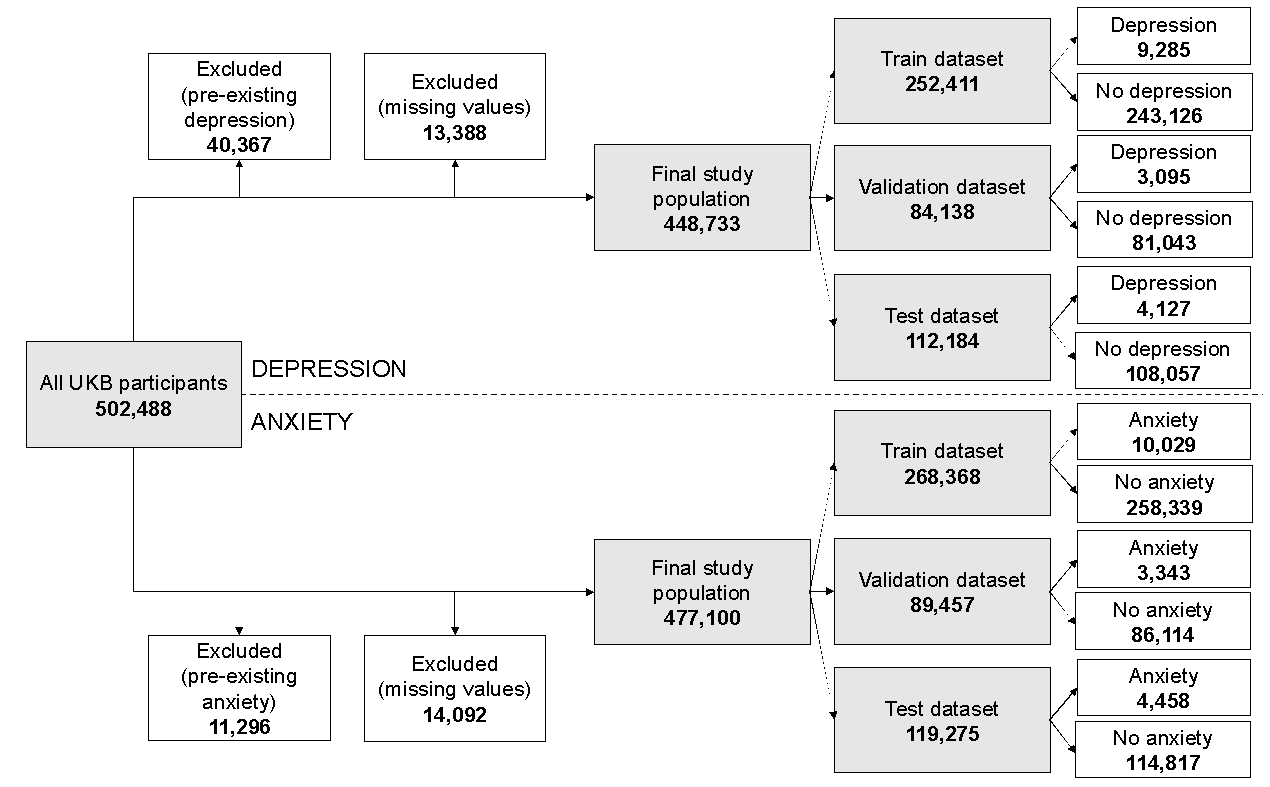


**Supplementary Figure 2: Flow diagram of participants through the study.** Separate arms for depression and anxiety are shown, including breakdown of outcome incidence in the train, validation and test datasets.


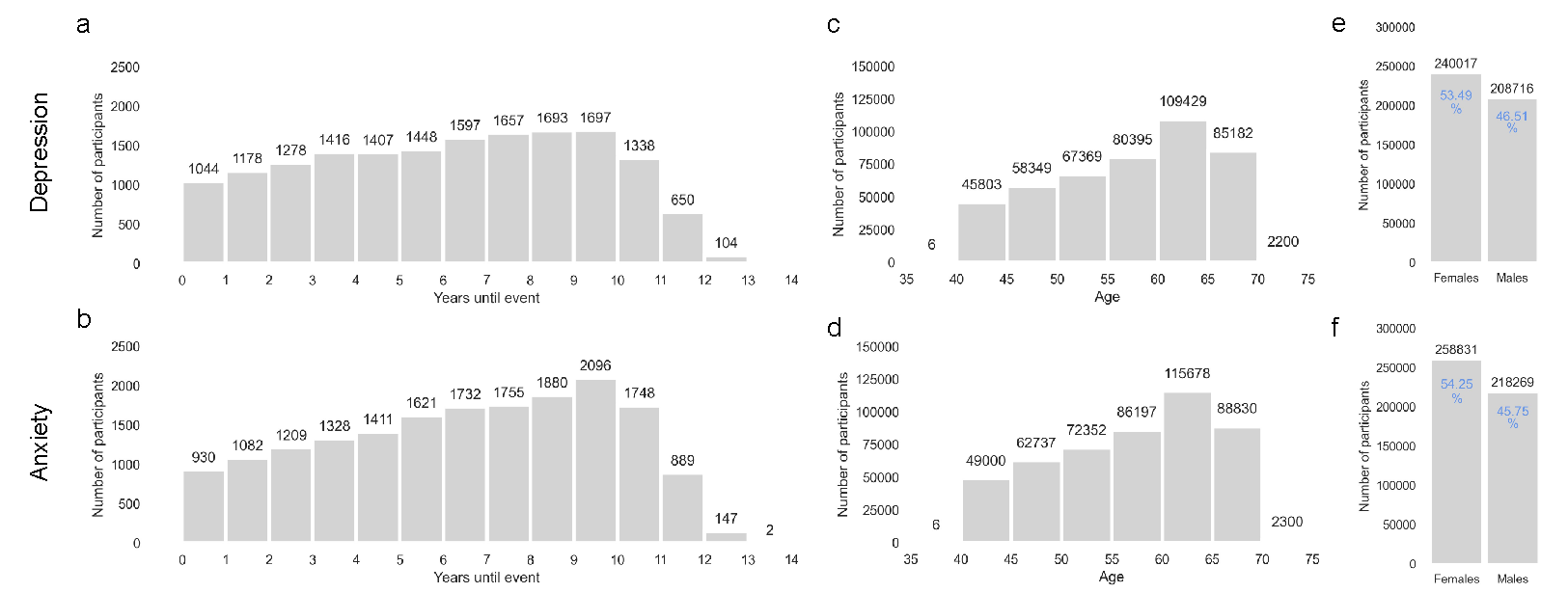


**Supplementary Figure 3**: **Distribution of time to event, age, and sex of the study participants.** Distribution of duration (in years) until the diagnosis of depression **(a)** or anxiety **(b).** Age distribution in the cohort used for modelling depression **(c)** and anxiety **(d)** Sex distribution among participants without pre-existing depression **(e)** and anxiety **(f).** Numbers above bars indicate the count of participants with the relevant characteristic. Blue numbers represent the percentage of total participants.


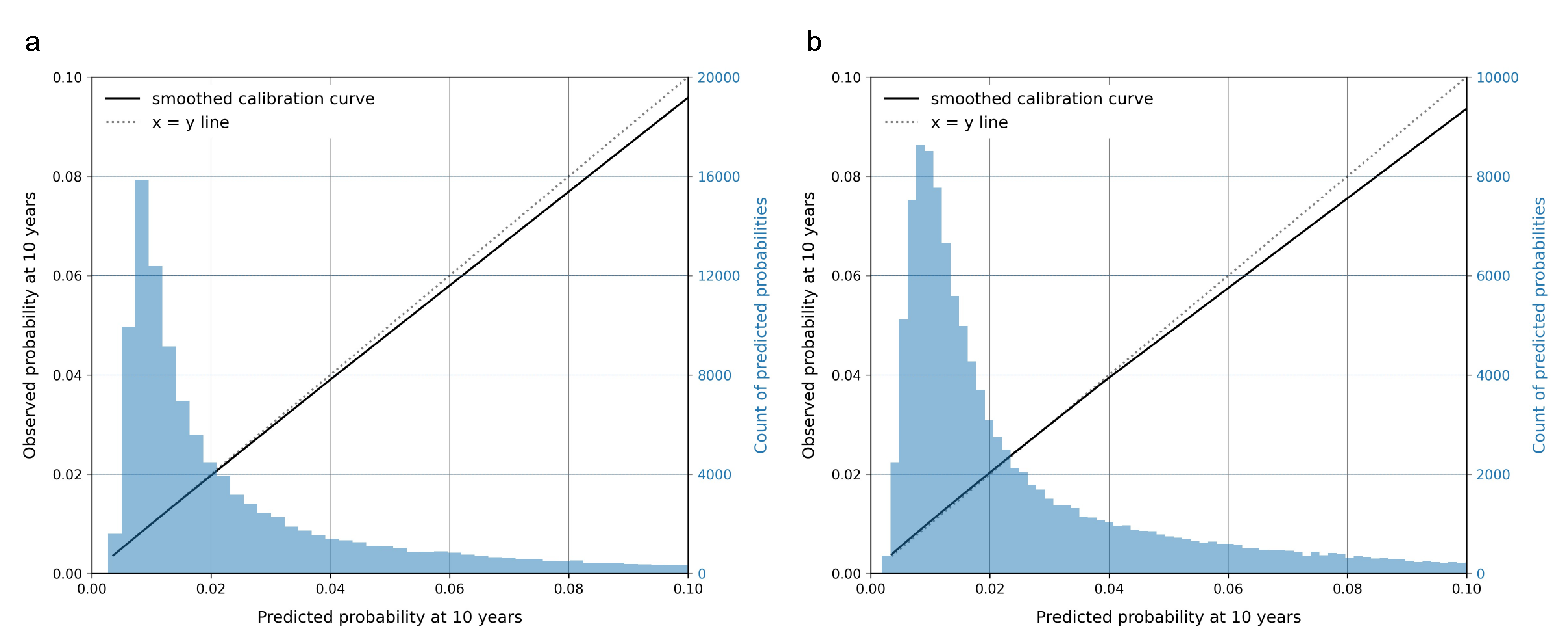


**Supplementary Figure 4: Calibration plots for 10-year probabilities of developing depression (a) and anxiety (b).** Results are shown for the final reduced Cox models evaluated on the test dataset. Smoothed calibration curve is shown along with the perfect calibration curve (x=y line). Histogram of the predicted probabilities of developing depression/anxiety for the participants in the test dataset are shown in blue.

**Supplementary Table 1: List of UK Biobank fields excluded from the input space due to potential label leaking.**

| **UK Biobank field number** | **Field name** |
| --- | --- |
| 4609 | Longest period of depression |
| 4620 | Number of depression episodes |
| 20123 | Single episode of probable major depression |
| 20124 | Probable recurrent major depression (moderate) |
| 20125 | Probable recurrent major depression (severe) |
| 20126 | Bipolar and major depression status |
| 20127 | Neuroticism score |
| 20499 | Ever sought or received professional help for mental distress |
| 20500 | Ever suffered mental distress preventing usual activities |
| 20544 | Mental health problems ever diagnosed by a professional |

**Supplementary Table 2: Summary of all UK Biobank input variables used in the study.** The variable name consists of name_UK-Biobank-field-number and original data completeness is shown. Method of pre-processing data before modelling is described in detail, along with the resulting number of input features arising from each original field for the depression (DEP) and anxiety (ANX) models.

| **Variable name _UK Biobank field number** | **Data complete-ness (%)** | **Processing pipeline** | **No. fields** |
| --- | --- | --- | --- |
| AbleToConfide_2110 | 99.62 | One-hot encoded,  dropped category”-1"  in pre-processing of data for both models due to low occurrence | 7 |
| AgeAtRecruitment_21022 | 100 | Standardised by removing the mean and scaling to unit variance, participants with missing values excluded | 1 |
| AlcoholIntakeFrequency._1558 | 99.82 | One-hot encoded | 7 |
| AverageTotalHouseholdIncomeBeforeTax_738 | 98.8 | One-hot encoded,  dropped category "-1"  in pre-processing of data for both models due to low occurrence | 6 |
| AverageWeeklyIntakeOfOtherAlcoholicDrinks_5364 | 23.1 | Dropped from both models due to high number of missing values | 0 |
| BeefIntake_1369 | 99.62 | One-hot encoded,  dropped categories “-1”, “-3” and "5"  in pre-processing of data for both models due to low occurrence | 5 |
| BipolarDisorderStatus_20122 | 42.66 | One-hot encoded | 2 |
| BodyMassIndex_21001 | 99.38 | Standardised by removing the mean and scaling to unit variance, participants with missing values excluded | 1 |
| CancerDiagnosedByDoctor_2453 | 99.81 | One-hot encoded,  dropped categories “-1" and “-3” in pre-processing of data for both models due to low occurrence | 2 |
| CheeseIntake_1408 | 97.24 | One-hot encoded,  dropped categories “-1" and “-3” in pre-processing of data for both models due to low occurrence | 6 |
| CoffeeIntake_1498 | 99.62 | Values of "-10" were converted to 0.5 (meaning is less than 1), missing values and values "-1" and "-3" were imputed with the mean, standardised by removing the mean and scaling to unit variance, participants with missing values excluded | 1 |
| CookedVegetableIntake_1289 | 99.63 | Values of "-10" were converted to 0.5 (meaning is less than 1), missing values and values "-1" and "-3" were imputed with the mean, standardised by removing the mean and scaling to unit variance, participants with missing values excluded | 1 |
| CurrentEmploymentStatus_6142 | 99.8 | One-hot encoded | 9 |
| CurrentTobaccoSmoking_1239 | 99.82 | One-hot encoded, dropped category “-3"  in pre-processing of data for both models due to low occurrence | 3 |
| DateF00FirstReported_130836 | 100 | Merged into a single field "any_mental_issue", converted into a binary field (1 if any of these diagnoses was present before the date of assessment)  **Note:** DateF32FirstReported_130894 was used as an outcome in the depression model, DateF41FirstReported_130906 was an outcome in the anxiety model and therefore not used as inputs in the respective models. | 1 |
| DateF01FirstReported_130838 |  |  |  |
| DateF02FirstReported_130840 |  |  |  |
| DateF03FirstReported_130842 |  |  |  |
| DateF04FirstReported_130844 |  |  |  |
| DateF05FirstReported_130846 |  |  |  |
| DateF06FirstReported_130848 |  |  |  |
| DateF07FirstReported_130850 |  |  |  |
| DateF09FirstReported_130852 |  |  |  |
| DateF10FirstReported_130854 |  |  |  |
| DateF11FirstReported_130856 |  |  |  |
| DateF12FirstReported_130858 |  |  |  |
| DateF13FirstReported_130860 |  |  |  |
| DateF14FirstReported_130862 |  |  |  |
| DateF15FirstReported_130864 |  |  |  |
| DateF16FirstReported_130866 |  |  |  |
| DateF17FirstReported_130868 |  |  |  |
| DateF18FirstReported_130870 |  |  |  |
| DateF19FirstReported_130872 |  |  |  |
| DateF20FirstReported_130874 |  |  |  |
| DateF21FirstReported_130876 |  |  |  |
| DateF22FirstReported_130878 |  |  |  |
| DateF23FirstReported_130880 |  |  |  |
| DateF24FirstReported_130882 |  |  |  |
| DateF25FirstReported_130884 |  |  |  |
| DateF28FirstReported_130886 |  |  |  |
| DateF29FirstReported_130888 |  |  |  |
| DateF30FirstReported_130890 |  |  |  |
| DateF31FirstReported_130892 |  |  |  |
| DateF32FirstReported_130894 |  |  |  |
| DateF34FirstReported_130898 |  |  |  |
| DateF38FirstReported_130900 |  |  |  |
| DateF39FirstReported_130902 |  |  |  |
| DateF40FirstReported_130904 |  |  |  |
| DateF41FirstReported_130906 |  |  |  |
| DateF42FirstReported_130908 |  |  |  |
| DateF43FirstReported_130910 |  |  |  |
| DateF44FirstReported_130912 |  |  |  |
| DateF45FirstReported_130914 |  |  |  |
| DateF48FirstReported_130916 |  |  |  |
| DateF50FirstReported_130918 |  |  |  |
| DateF51FirstReported_130920 |  |  |  |
| DateF52FirstReported_130922 |  |  |  |
| DateF53FirstReported_130924 |  |  |  |
| DateF54FirstReported_130926 |  |  |  |
| DateF55FirstReported_130928 |  |  |  |
| DateF59FirstReported_130930 |  |  |  |
| DateF60FirstReported_130932 |  |  |  |
| DateF61FirstReported_130934 |  |  |  |
| DateF62FirstReported_130936 |  |  |  |
| DateF63FirstReported_130938 |  |  |  |
| DateF64FirstReported_130940 |  |  |  |
| DateF65FirstReported_130942 |  |  |  |
| DateF66FirstReported_130944 |  |  |  |
| DateF68FirstReported_130946 |  |  |  |
| DateF69FirstReported_130948 |  |  |  |
| DateF70FirstReported_130950 |  |  |  |
| DateF71FirstReported_130952 |  |  |  |
| DateF72FirstReported_130954 |  |  |  |
| DaytimeDozing/Sleeping_1220 | 99.82 | One-hot encoded,  dropped categories “-1" and "3"  in pre-processing of data for both models due to low occurrence | 4 |
| DiabetesDiagnosedByDoctor_2443 | 99.81 | One-hot encoded,  dropped categories “-1" and “-3"  in pre-processing of data for both models due to low occurrence | 2 |
| DifferenceInMobilePhoneUseComparedToTwoYearsPreviously_1140 | 84.08 | One-hot encoded,  dropped categories “-1" and “-3"  in pre-processing of data for both models due to low occurrence | 4 |
| EthnicBackground_21000 | 99.82 | One-hot encoded,  dropped categories “-1", "2", "3", "4", "2002", "3003" and "4003"  in pre-processing of data for both models due to low occurrence | 7 |
| EverDepressedForAWholeWeek_4598 | 42.66 | One-hot encoded,  dropped category “-1"  in pre-processing of data for both models due to low occurrence | 3 |
| EverHighlyIrritable/ArgumentativeFor2Days_4653 | 42.66 | One-hot encoded, dropped category “-1"  in pre-processing of data for both models due to low occurrence | 3 |
| EverManic/HyperFor2Days_4642 | 42.66 | One-hot encoded,  dropped category “-1"  in pre-processing of data for both models due to low occurrence | 3 |
| EverUnenthusiastic/DisinterestedForAWholeWeek_4631 | 42.66 | One-hot encoded,  dropped category “-1"  in pre-processing of data for both models due to low occurrence | 3 |
| FamilyRelationshipSatisfaction_4559 | 42.66 | One-hot encoded,  dropped category “-1"  in pre-processing of data for both models due to low occurrence | 7 |
| Fed-UpFeelings_1960 | 99.62 | One-hot encoded,  dropped category “-1"  in pre-processing of data for both models due to low occurrence | 3 |
| FinancialSituationSatisfaction_4581 | 42.66 | One-hot encoded,  dropped category “-1"  in pre-processing of data for both models due to low occurrence | 7 |
| Fractured/BrokenBonesInLast5Years_2463 | 99.62 | One-hot encoded,  dropped categories “-1" and “-3"  in pre-processing of data for both models due to low occurrence | 2 |
| FrequencyOfDepressedMoodInLast2Weeks_2050 | 99.62 | One-hot encoded,  dropped category “-1"  in pre-processing of data for both models due to low occurrence | 5 |
| FrequencyOfFriend/FamilyVisits_1031 | 98.88 | One-hot encoded,  dropped category “-1"  in pre-processing of data for both models due to low occurrence | 8 |
| FrequencyOfSolarium/SunlampUse_2277 | 98.87 | Standardised by removing the mean and scaling to unit variance, participants with missing values excluded | 1 |
| FrequencyOfTenseness/RestlessnessInLast2Weeks_2070 | 99.62 | One-hot encoded,  dropped category “-1"  in pre-processing of data for both models due to low occurrence | 5 |
| FrequencyOfTiredness/LethargyInLast2Weeks_2080 | 99.62 | One-hot encoded,  dropped category “-1"  in pre-processing of data for both models due to low occurrence | 5 |
| FrequencyOfUnenthusiasm/DisinterestInLast2Weeks_2060 | 99.62 | One-hot encoded,  dropped category “-1"  in pre-processing of data for anxiety both models to low occurrence | 5 |
| FreshFruitIntake_1309 | 99.63 | Values of "-10" were converted to 0.5 (meaning is less than 1), missing values and values "-1" and "-3" were imputed with the mean, standardised by removing the mean and scaling to unit variance, participants with missing values excluded | 1 |
| FriendshipsSatisfaction_4570 | 42.66 | One-hot encoded,  dropped categories “-1" and "6"  in pre-processing of data for both models due to low occurrence | 6 |
| GettingUpInMorning_1170 | 99.07 | One-hot encoded,  dropped categories “-1" and “-3"  in pre-processing of data for both models due to low occurrence | 4 |
| GuiltyFeelings_2030 | 99.62 | One-hot encoded,  dropped category “-1"  in pre-processing of data for both models due to low occurrence | 3 |
| HadMajorOperations_2415 | 45.41 | Sex-specific fields merged, dropped categories “-1" and “-3” in pre-processing of data for both models due to low occurrence | 5 |
| HadOtherMajorOperations_2844 | 45.8 |  |  |
| Happiness_4526 | 42.66 | One-hot encoded,  dropped categories “-3”, “-1” and "6"  in pre-processing of data for both models due to low occurrence | 5 |
| HealthSatisfaction_4548 | 42.66 | One-hot encoded,  dropped category “-3” and “-1”  in pre-processing of data for both models due to low occurrence | 6 |
| HearingDifficulty/Problems_2247 | 98.87 | One-hot encoded,  dropped categories "-1" and "99"  in pre-processing of data for both models due to low occurrence | 3 |
| HipCircumference_49 | 99.56 | Standardised by removing the mean and scaling to unit variance, participants with missing values excluded | 1 |
| Illness,Injury,Bereavement,StressInLast2Years_6145 | 99.8 | One-hot encoded | 8 |
| IllnessesOfFather_20107 | 99.8 | One-hot encoded, dropped category "-23" in pre-processing of data for both models due to low occurrence | 16 |
| IllnessesOfMother_20110 | 99.8 | One-hot encoded,  dropped categories "-23" and "-13"  in pre-processing of data for both models due to low occurrence | 15 |
| IllnessesOfSiblings_20111 | 99.8 | One-hot encoded, dropped category "-23" in pre-processing of data for both models due to low occurrence | 17 |
| Irritability_1940 | 99.62 | One-hot encoded,  dropped category “-1"  in pre-processing of data for both models due to low occurrence | 3 |
| JobInvolvesHeavyManualOrPhysicalWork_816 | 99.83 | One-hot encoded | 4 |
| JobInvolvesMainlyWalkingOrStanding_806 | 99.83 | One-hot encoded,  dropped categories “-3” and "-1"  in pre-processing of data for both models due to low occurrence | 4 |
| JobInvolvesShiftWork_826 | 99.83 | One-hot encoded,  dropped categories “-3” and “-1”  in pre-processing of data for both models due to low occurrence | 4 |
| Lamb/MuttonIntake_1379 | 99.62 | One-hot encoded,  dropped categories “-3”, "-1", "4" and "5"  in pre-processing of data for both models due to low occurrence | 4 |
| LegPainOnWalking_4728 | 42.66 | One-hot encoded, dropped categories “-3” and "-1"  in pre-processing of data for both models due to low occurrence | 2 |
| Leisure/SocialActivities_6160 | 99.8 | One-hot encoded | 7 |
| LengthOfLongestManic/IrritableEpisode_5663 | 42.66 | One-hot encoded,  dropped categories “-3” and “-1”  in pre-processing of data for both models due to low occurrence | 3 |
| LengthOfMobilePhoneUse_1110 | 99.63 | One-hot encoded,  dropped category “-1"  in pre-processing of data for both models due to low occurrence | 6 |
| Loneliness,Isolation_2020 | 99.62 | One-hot encoded,  dropped category “-1"  in pre-processing of data for both models due to low occurrence | 3 |
| Manic/HyperSymptoms_6156 | 42.66 | One-hot encoded | 6 |
| MeanTimeToCorrectlyIdentifyMatches_20023 | 98.84 | Standardised by removing the mean and scaling to unit variance, participants with missing values excluded | 1 |
| Miserableness_1930 | 99.62 | One-hot encoded,  dropped category “-1"  in pre-processing of data for both models due to low occurrence | 3 |
| MoodSwings_1920 | 99.62 | One-hot encoded,  dropped category “-1"  in pre-processing of data for both models due to low occurrence | 3 |
| Morning/EveningPerson_1180 | 99.07 | One-hot encoded,  dropped category “-1"  in pre-processing of data for both models  due to low occurrence | 5 |
| Mouth/TeethDentalProblems_6149 | 99.8 | One-hot encoded | 8 |
| NapDuringDay_1190 | 99.82 | One-hot encoded,  dropped category “-1"  in pre-processing of data for both models due to low occurrence | 4 |
| NervousFeelings_1970 | 99.62 | One-hot encoded,  dropped category “-1"  in pre-processing of data for both models due to low occurrence | 3 |
| NumberOfCigarettesCurrentlySmokedDaily_3456 | 7.26 | Dropped from both models due to high number of missing values | 0 |
| NumberOfOperations,Self-Reported_136 | 99.63 | Standardised by removing the mean and scaling to unit variance, participants with missing values excluded | 1 |
| NumberOfSelf-ReportedCancers_134 | 99.83 | Standardised by removing the mean and scaling to unit variance, participants with missing values excluded | 1 |
| OtherSeriousMedicalCondition/DisabilityDiagnosedByDoctor_2473 | 99.62 | One-hot encoded,  dropped category “-1"  in pre-processing of data for both models due to low occurrence | 3 |
| OverallHealthRating_2178 | 99.82 | One-hot encoded,  dropped categories “-3” and “-1"  in pre-processing of data for both models due to low occurrence | 4 |
| PackYearsOfSmoking_20161 | 30.04 | Dropped from both models due to high number of missing values | 0 |
| PainTypeExperiencedInLastMonth_6159 | 99.8 | One-hot encoded | 10 |
| PastTobaccoSmoking_1249 | 92.01 | One-hot encoded | 5 |
| PlaysComputerGames_2237 | 99.62 | One-hot encoded | 4 |
| PorkIntake_1389 | 99.62 | One-hot encoded,  dropped categories “-3”, “-1”, "4" and "5"  in pre-processing of data for both models due to low occurrence | 4 |
| PoultryIntake_1359 | 99.62 | One-hot encoded,  dropped categories “-3” and “-1"  in pre-processing of data for both models due to low occurrence | 6 |
| ProcessedMeatIntake_1349 | 99.62 | One-hot encoded,  dropped categories “-3” and “-1"  in pre-processing of data for both models due to low occurrence | 6 |
| Qualifications_6138 | 99.8 | One-hot encoded | 8 |
| RiskTaking_2040 | 99.62 | One-hot encoded,  dropped category “-1"  in pre-processing of data for both models due to low occurrence | 3 |
| Salad/RawVegetableIntake_1299 | 99.63 | Values of "-10" were converted to 0.5 (meaning is less than 1), missing values and values "-1" and "-3" were imputed with the mean, standardised by removing the mean and scaling to unit variance, participants with missing values excluded | 1 |
| SaltAddedToFood_1478 | 99.62 | One-hot encoded, dropped category “-3”  in pre-processing of data for both models due to low occurrence | 4 |
| SeenAPsychiatristForNerves,Anxiety,TensionOrDepression_2100 | 99.62 | One-hot encoded,  dropped category “-1"  in pre-processing of data for both models due to low occurrence | 3 |
| SeenDoctorForNerves,Anxiety,TensionOrDepression_2090 | 99.62 | One-hot encoded,  dropped category “-1"  in pre-processing of data for both models due to low occurrence | 3 |
| Sensitivity/HurtFeelings_1950 | 99.62 | One-hot encoded,  dropped category “-1"  in pre-processing of data for both models due to low occurrence | 3 |
| SeverityOfManic/IrritableEpisodes_5674 | 42.66 | One-hot encoded,  dropped category “-1"  in pre-processing of data for both models due to low occurrence | 3 |
| Sex_31 | 100 | One-hot encoded | 2 |
| SkinColour_1717 | 99.62 | One-hot encoded,  dropped category “-1"  in pre-processing of data for both models due to low occurrence | 7 |
| SleepDuration_1160 | 99.82 | Standardised by removing the mean and scaling to unit variance, participants with missing values excluded | 1 |
| Sleeplessness/Insomnia_1200 | 99.82 | One-hot encoded | 4 |
| SmokingStatus_20116 | 99.82 | One-hot encoded | 4 |
| Snoring_1210 | 99.82 | One-hot encoded,  dropped category “-1"  in pre-processing of data for both models due to low occurrence | 3 |
| StandingHeight_50 | 99.49 | Standardised by removing the mean and scaling to unit variance, participants with missing values excluded | 1 |
| SufferFrom'Nerves'_2010 | 99.62 | One-hot encoded,  dropped category “-1"  in pre-processing of data for both models due to low occurrence | 3 |
| TeaIntake_1488 | 99.62 | Values of "-10" were converted to 0.5 (meaning is less than 1), missing values and values "-1" and "-3" were imputed with the mean, standardised by removing the mean and scaling to unit variance, participants with missing values excluded | 1 |
| Tense/'HighlyStrung'_1990 | 99.62 | One-hot encoded,  dropped category “-1"  in pre-processing of data for both models due to low occurrence | 3 |
| TimeSpendOutdoorsInSummer_1050 | 98.88 | Standardised by removing the mean and scaling to unit variance, participants with missing values excluded | 1 |
| TimeSpentOutdoorsInWinter_1060 | 98.88 | Standardised by removing the mean and scaling to unit variance, participants with missing values excluded | 1 |
| Tinnitus_4803 | 34.27 | One-hot encoded,  dropped categories “-3” and “-1"  in pre-processing of data for both models due to low occurrence | 5 |
| UseOfSun/UvProtection_2267 | 98.87 | One-hot encoded,  dropped category “-1"  in pre-processing of data for both models due to low occurrence | 6 |
| WaistCircumference_48 | 99.57 | Standardised by removing the mean and scaling to unit variance, participants with missing values excluded | 1 |
| WaterIntake_1528 | 99.62 | Values of "-10" were converted to 0.5 (meaning is less than 1), missing values and values "-1" and "-3" were imputed with the mean, standardised by removing the mean and scaling to unit variance, participants with missing values excluded | 1 |
| WeeklyUsageOfMobilePhoneInLast3Months_1120 | 84.08 | One-hot encoded,  dropped categories “-3” and “-1"  in pre-processing of data for both models due to low occurrence | 6 |
| Weight_21002 | 99.45 | Standardised by removing the mean and scaling to unit variance, participants with missing values excluded | 1 |
| Work/JobSatisfaction_4537 | 34.3 | One-hot encoded,  dropped category “-1"  in pre-processing of data for both models due to low occurrence | 8 |
| Worrier/AnxiousFeelings_1980 | 99.62 | One-hot encoded,  dropped category “-1"  in pre-processing of data for both models due to low occurrence | 3 |
| WorryTooLongAfterEmbarrassment_2000 | 99.62 | One-hot encoded,  dropped category “-1"  in pre-processing of data for both models due to low occurrence | 3 |
| **TOTAL:** |  |  | **429** |

**Supplementary Table 3: Characteristics of participants excluded from the depression dataset due to missing continuous features.** Last column shows p-value after comparing the incident depression group with the non-depression group. Comparisons were performed using the Chi-squared test for categories and Kruskal-Wallis test for continuous variables.

|  | n (%) | | | P-Value (adjusted) |
| --- | --- | --- | --- | --- |
|  | All participants | Excluded | Included |  |
| n | 462121 | 13388 | 448733 |  |
| Incident depression, n (%) | 17253  (3.73) | 746  (5.57) | 16507  (3.68) | <0.001 |
| Time-to-event (outcome/censoring), median [Q1,Q3] | 11.56 [10.80,12.28] | 11.79 [10.60,14.31] | 11.56 [10.80,12.27] | <0.001 |
| A worrier, n (%) | 244415  (52.89) | 6046  (45.16) | 238369  (53.12) | <0.001 |
| Able to confide almost daily, n (%) | 241140  (52.18) | 5294  (39.54) | 235846  (52.56) | <0.001 |
| Age, median [Q1,Q3] | 58.00 [50.00,63.00] | 58.00 [50.00,64.00] | 58.00 [50.00,63.00] | 1 |
| Annual household income less than £18,000, n (%) | 85211  (18.44) | 2618  (19.55) | 82593  (18.41) | 0.029 |
| Annual household income over £100,000, n (%) | 21979  (4.76) | 282  (2.11) | 21697  (4.84) | <0.001 |
| CSEs or equivalent qualification, n (%) | 58607  (12.68) | 634  (4.74) | 57973  (12.92) | <0.001 |
| Does not have university degree, A/O levels, GCSE/CSE or professional qualifications, n (%) | 77305  (16.73) | 2554  (19.08) | 74751  (16.66) | <0.001 |
| Experienced financial difficulties in the past 2 years, n (%) | 51772  (11.20) | 1208  (9.02) | 50564  (11.27) | <0.001 |
| Experienced serious illness, injury or assault to themselves in the last 2 years, n (%) | 40666  (8.80) | 1011  (7.55) | 39655  (8.84) | <0.001 |
| Experiences mood swings, n (%) | 191419  (41.42) | 5257  (39.27) | 186162  (41.49) | <0.001 |
| Fair self-rated overall health, n (%) | 92653  (20.05) | 3268  (24.41) | 89385  (19.92) | <0.001 |
| Felt depressed on several days over the past two weeks, n (%) | 74668  (16.16) | 1887  (14.09) | 72781  (16.22) | <0.001 |
| Has been depressed nearly every day over the past two weeks, n (%) | 5687  (1.23) | 340  (2.54) | 5347  (1.19) | <0.001 |
| Has been depressed or down for a whole week in the past, n (%) | 76560  (16.57) | 1438  (10.74) | 75122  (16.74) | <0.001 |
| Has not been unenthusiastic or disinterested for a whole week, n (%) | 100897  (21.83) | 2056  (15.36) | 98841  (22.03) | <0.001 |
| Has not felt tired in the last 2 weeks, n (%) | 216493  (46.85) | 4710  (35.18) | 211783  (47.20) | <0.001 |
| Has seen a GP for nerves, anxiety, tension or depression, n (%) | 131247  (28.40) | 3396  (25.37) | 127851  (28.49) | <0.001 |
| Has seen a psychiatrist for nerves, anxiety, tension or depression, n (%) | 38198  (8.27) | 1306  (9.76) | 36892  (8.22) | <0.001 |
| Height, median [Q1,Q3] | 168.00 [162.00,175.00] | 167.00 [160.00,174.00] | 168.00 [162.00,175.00] | <0.001 |
| Manic/irritable episodes ended up in needing treatment or caused problems in life, n (%) | 5760  (1.25) | 171  (1.28) | 5589  (1.25) | 1 |
| More a 'morning' than 'evening' person, n (%) | 146137  (31.62) | 2296  (17.15) | 143841  (32.05) | <0.001 |
| Mother diagnosed with severe depression, n (%) | 24888  (5.39) | 577  (4.31) | 24311  (5.42) | <0.001 |
| Never drinks alcohol, n (%) | 35658  (7.72) | 2077  (15.51) | 33581  (7.48) | <0.001 |
| Never smoked, n (%) | 186779  (40.42) | 5490  (41.01) | 181289  (40.40) | 1 |
| Not tense or highly strung, n (%) | 373194  (80.76) | 8326  (62.19) | 364868  (81.31) | <0.001 |
| Number of operations, median [Q1,Q3] | 1.00  [1.00,2.00] | 1.00  [0.00,2.00] | 1.00  [1.00,2.00] | <0.001 |
| Often feels fed-up, n (%) | 171468  (37.10) | 4524  (33.79) | 166944  (37.20) | <0.001 |
| Poor self-rated overall health, n (%) | 17407  (3.77) | 1102  (8.23) | 16305  (3.63) | <0.001 |
| Previous smoker, n (%) | 158946  (34.39) | 3768  (28.14) | 155178  (34.58) | <0.001 |
| Sex, n (%) | 215185  (46.56) | 6469  (48.32) | 208716  (46.51) | 0.001 |
| Sometimes feels miserable for no reason, n (%) | 180131  (38.98) | 4399  (32.86) | 175732  (39.16) | <0.001 |
| Suffers from nerves, n (%) | 82810  (17.92) | 2274  (16.99) | 80536  (17.95) | 0.167 |
| Their feelings are easily hurt, n (%) | 239762  (51.88) | 5862  (43.79) | 233900  (52.12) | <0.001 |
| Tried smoking just once or twice in the past, n (%) | 67662  (14.64) | 1465  (10.94) | 66197  (14.75) | <0.001 |
| Unable to work because of sickness or disability, n (%) | 14407  (3.12) | 1075  (8.03) | 13332  (2.97) | <0.001 |
| Water intake, median [Q1,Q3] | 2.00  [1.00,4.00] | 3.00  [1.00,4.00] | 2.00  [1.00,4.00] | <0.001 |

**Supplementary Table 4: Characteristics of participants excluded from the anxiety dataset due to missing continuous features.** Last column shows p-value after comparing the incident depression group with the non-depression group. Comparisons were performed using the Chi-squared test for categories and Kruskal-Wallis test for continuous variables.

|  | n (%) | | | P-Value (adjusted) |
| --- | --- | --- | --- | --- |
|  | All participants | Excluded | Included |  |
| n | 491192 | 14092 | 477100 |  |
| Incident anxiety, n (%) | 18519  (3.77) | 689  (4.89) | 17830  (3.74) | <0.001 |
| Time-to-event (outcome/censoring), median [Q1,Q3] | 11.57 [10.80,12.30] | 11.79 [10.61,13.78] | 11.56 [10.80,12.29] | <0.001 |
| Age, median [Q1,Q3] | 58.00 [50.00,63.00] | 58.00 [50.00,64.00] | 58.00 [50.00,63.00] | 1.000 |
| Annual household income between £31,000 and £51,999, n (%) | 108655  (22.12) | 1262  (8.96) | 107393  (22.51) | <0.001 |
| Annual household income between £52,000 and £100,000, n (%) | 84935  (17.29) | 940  (6.67) | 83995  (17.61) | <0.001 |
| Annual household income over £100,000, n (%) | 22727  (4.63) | 291  (2.07) | 22436  (4.70) | <0.001 |
| Body mass index, median [Q1,Q3] | 26.74 [24.13,29.90] | 27.11 [24.36,30.46] | 26.73 [24.13,29.88] | <0.001 |
| Close partner or relative complains about their snoring, n (%) | 169581  (34.52) | 4215  (29.91) | 165366  (34.66) | <0.001 |
| College or university degree, n (%) | 157873  (32.14) | 2131  (15.12) | 155742  (32.64) | <0.001 |
| Does not know the illnesses of father, n (%) | 33583  (6.84) | 1284  (9.11) | 32299  (6.77) | <0.001 |
| Eats beef 2-4 times a week, n (%) | 55165  (11.23) | 1416  (10.05) | 53749  (11.27) | <0.001 |
| Excellent self-rated overall health, n (%) | 80918  (16.47) | 1628  (11.55) | 79290  (16.62) | <0.001 |
| Experiences mood swings, n (%) | 214338  (43.64) | 5773  (40.97) | 208565  (43.72) | <0.001 |
| Fair self-rated overall health, n (%) | 101991  (20.76) | 3482  (24.71) | 98509  (20.65) | <0.001 |
| Felt tense or restless nearly every day in the last 2 weeks, n (%) | 8596  (1.75) | 442  (3.14) | 8154  (1.71) | <0.001 |
| Finds getting up in the morning fairly easy, n (%) | 241419  (49.15) | 4064  (28.84) | 237355  (49.75) | <0.001 |
| Finds getting up in the morning very easy, n (%) | 157724  (32.11) | 3028  (21.49) | 154696  (32.42) | <0.001 |
| Has been highly irritable or argumentative for two days in the past, n (%) | 133114  (27.10) | 2712  (19.24) | 130402  (27.33) | <0.001 |
| Has been unenthusiastic or disinterested for a whole week, n (%) | 57138  (11.63) | 1189  (8.44) | 55949  (11.73) | <0.001 |
| Has fractured bones in the last 5 years, n (%) | 46014  (9.37) | 1234  (8.76) | 44780  (9.39) | 0.589 |
| Has not felt depressed at all over the past two weeks, n (%) | 358594  (73.00) | 7962  (56.50) | 350632  (73.49) | <0.001 |
| Has not seen a GP for nerves, anxiety, tension or depression, n (%) | 325721  (66.31) | 7850  (55.71) | 317871  (66.63) | <0.001 |
| Has not seen a psychiatrist for nerves, anxiety, tension or depression, n (%) | 433567  (88.27) | 10219  (72.52) | 423348  (88.73) | <0.001 |
| Headache experienced in the last month, n (%) | 99516  (20.26) | 2740  (19.44) | 96776  (20.28) | 0.728 |
| Job never or rarely involves heavy manual/physical work, n (%) | 182641  (37.18) | 3413  (24.22) | 179228  (37.57) | <0.001 |
| Job sometimes involves heavy manual/physical work, n (%) | 60344  (12.29) | 1376  (9.76) | 58968  (12.36) | <0.001 |
| Longest manic/irritable episode lasted 2-7 days, n (%) | 16478  (3.35) | 337  (2.39) | 16141  (3.38) | <0.001 |
| Never feels miserable for no reason, n (%) | 277266  (56.45) | 6744  (47.86) | 270522  (56.70) | <0.001 |
| Never/rarely takes a nap during the day, n (%) | 275462  (56.08) | 6466  (45.88) | 268996  (56.38) | <0.001 |
| Not a nervous person, n (%) | 365864  (74.48) | 8520  (60.46) | 357344  (74.90) | <0.001 |
| Not a worrier, n (%) | 209258  (42.60) | 5112  (36.28) | 204146  (42.79) | <0.001 |
| Not an irritable person, n (%) | 337176  (68.64) | 8217  (58.31) | 328959  (68.95) | <0.001 |
| Number of operations, median [Q1,Q3] | 1.00  [1.00,2.00] | 1.00  [0.00,2.00] | 1.00  [1.00,3.00] | <0.001 |
| Pain all over the body experienced in the last month, n (%) | 8422  (1.71) | 464  (3.29) | 7958  (1.67) | <0.001 |
| Pain in leg when walking, n (%) | 38151  (7.77) | 1413  (10.03) | 36738  (7.70) | <0.001 |
| Poor self-rated overall health, n (%) | 21507  (4.38) | 1292  (9.17) | 20215  (4.24) | <0.001 |
| Prefers not to answer about the illnesses of father, n (%) | 625  (0.13) | 79  (0.56) | 546  (0.11) | <0.001 |
| Sex, n (%) | 225035  (45.81) | 6766  (48.01) | 218269  (45.75) | <0.001 |
| Sibling diagnosed with severe depression, n (%) | 27086  (5.51) | 649  (4.61) | 26437  (5.54) | <0.001 |
| Sibling not diagnosed with any of the diseases, n (%) | 257929  (52.51) | 5905  (41.90) | 252024  (52.82) | <0.001 |
| Smokes tobacco on most or all days, n (%) | 37911  (7.72) | 1392  (9.88) | 36519  (7.65) | <0.001 |
| Sometimes has trouble falling asleep or wakes up at night, n (%) | 233703  (47.58) | 6247  (44.33) | 227456  (47.67) | <0.001 |
| Stomach or abdominal pain experienced in the last month, n (%) | 42303  (8.61) | 1297  (9.20) | 41006  (8.59) | 0.568 |
| Suffers from nerves, n (%) | 96501  (19.65) | 2596  (18.42) | 93905  (19.68) | 0.011 |
| Tense or highly strung, n (%) | 81383  (16.57) | 2494  (17.70) | 78889  (16.54) | 0.013 |
| Their feelings are easily hurt, n (%) | 261089  (53.15) | 6327  (44.90) | 254762  (53.40) | <0.001 |
| Usually has trouble falling asleep or wakes up at night, n (%) | 137018  (27.89) | 3609  (25.61) | 133409  (27.96) | <0.001 |
| Water intake, median [Q1,Q3] | 2.00  [1.00,4.00] | 3.00  [1.00,4.00] | 2.00  [1.00,4.00] | <0.001 |

**Supplementary Table 5:** Specifications of the python (v3.7.9) library versions used in this study. Example of training the DeepSurv model using the pycox library can be found here: <https://github.com/havakv/pycox/blob/60992d0f204cbaf232f2482834d81a838fe68b06/examples/cox-ph.ipynb>

| **Library** | **Version** |
| --- | --- |
| pandas | 1.1.0 |
| numpy | 1.17.0 |
| lifelines^A^ | 0.25.6 |
| scikit-learn | 0.23.1 |
| matplotlib | 3.2.1 |
| tableone | 0.7.9 |
| torch^B^ | 1.7.1 |
| optuna^C^ | 2.4.0 |
| pycox^D^ | 0.2.1 |
| torchtuples | 0.2.0 |

*^A^ Davidson-Pilon C, Kalderstam J, Jacobson N, Reed S, Kuhn B, Zivich P, Williamson M, AbdealiJK, Datta D, Fiore-Gartland A, Parij A, WIlson D, Gabriel, Moneda L, Moncada-Torres A, Stark K, Gadgil H, Jona, Singaravelan K, Besson L, Peña MS, Anton S, Klintberg A, GrowthJeff, Noorbakhsh J, Begun M, Kumar R, Hussey S, Seabold S, Golland D. CamDavidsonPilon/lifelines. 2020.*

*^B^ Paszke A, Gross S, Massa F, Lerer A, Bradbury J, Chanan G, et al. Pytorch: An imperative style, high-performance deep learning library. ArXiv Prepr ArXiv191201703. 2019;*

*^C^ Akiba T, Sano S, Yanase T, Ohta T, Koyama M. Optuna: A next-generation hyperparameter optimization framework. In: Proceedings of the 25th ACM SIGKDD international conference on knowledge discovery & data mining. 2019. p. 2623–31.*

*^D^ Kvamme H, Borgan Ø, Scheel I. Time-to-event prediction with neural networks and Cox regression. ArXiv Prepr ArXiv190700825. 2019;*

*^E^ Pölsterl S. scikit-survival: A Library for Time-to-Event Analysis Built on Top of scikit-learn. J Mach Learn Res. 2020;21(212):1–6.*

**Supplementary Table 6: DeepSurv hyper-parameter search space.**

| **Hyper-Parameter** | **Search Space** | **Optimal parameters for baseline depression model** | **Optimal parameters for baseline anxiety model** | **Optimal parameters for reduced depression model** | **Optimal parameters for reduced anxiety model** |
| --- | --- | --- | --- | --- | --- |
| Trial # | Total of 500 combinations | 271 | 199 | 173 | 117 |
| Activation | LeakyReLU [^48^](https://paperpile.com/c/iC6FLS/JMm7o), ReLU [^49^](https://paperpile.com/c/iC6FLS/x76KE) and SELU [^50^](https://paperpile.com/c/iC6FLS/O1s9T) | LeakyReLU | ReLU | LeakyReLU | ReLU |
| Categorical encoding | One Hot, Target Encoder, Weight Of Evidence | OneHot | OneHot | Weight Of Evidence | OneHot |
| Hidden Layers topology | 8, 32, 256, 32x32, 64x64, 128x128, 64x16, 256x32, 32x32x32, 64x64x64 | 64x64x64 | 32 | 32x32 | 32 |
| Drop-Out* [^51^](https://paperpile.com/c/iC6FLS/bRCyu) | [0, 0.9] | 0.6310 | 0.2619 | 0.8338 | 0.2619 |
| Weight-Decay* [^52^](https://paperpile.com/c/iC6FLS/1O0lL) | [0, 20] | 0.0421 | 0.0130 | 0.5532 | 0.0130 |
| Batch Normalization [^53^](https://paperpile.com/c/iC6FLS/ZWVom) | Yes/No | Yes | Yes | Yes | Yes |
| Optimizer | Stochastic Gradient Descent, Adam[^41^](https://paperpile.com/c/iC6FLS/QyLyz) | SGD | Adam | SGD | Adam |
| Momentum* | [0,1] | 0.9992 | - | 0.8250 | - |
| Learning Rate | Log distribution on [1e-5, 1] | 1.4171e-05 | 6.8803e-04 | 8.1295e-05 | 6.8804e-04 |

**Uniform distributions*

**Supplementary Table 7: Comparison of train+validation and test depression datasets.** Final set of variables after feature selection is shown. Last column shows p-value after comparing the incident depression group with the non-depression group. Comparisons were performed using the Chi-squared test for categories and Kruskal-Wallis test for continuous variables.

|  | n (%) | | | P-Value (adjusted) |
| --- | --- | --- | --- | --- |
|  | Full dataset | Train + validation | Test |  |
| n | 448733 | 336549 | 112184 |  |
| Incident depression, n (%) | 16507  (3.68) | 12380  (3.68) | 4127  (3.68) | 1 |
| Time-to-event (outcome/censoring), median [Q1,Q3] | 11.56 [10.80,12.27] | 11.56 [10.80,12.27] | 11.56 [10.80,12.27] | 1 |
| Able to confide almost daily, n (%) | 235846  (52.56) | 176910  (52.57) | 58936  (52.54) | 1 |
| Never drinks alcohol, n (%) | 33581  (7.48) | 25079  (7.45) | 8502  (7.58) | 1 |
| Annual household income less than ¬£18,000, n (%) | 82593  (18.41) | 61766  (18.35) | 20827  (18.57) | 1 |
| Annual household income over ¬£100,000, n (%) | 21697  (4.84) | 16248  (4.83) | 5449  (4.86) | 1 |
| Unable to work because of sickness or disability, n (%) | 13332  (2.97) | 10006  (2.97) | 3326  (2.96) | 1 |
| Has been depressed or down for a whole week in the past, n (%) | 75122  (16.74) | 56146  (16.68) | 18976  (16.92) | 1 |
| Has not been unenthusiastic orr disinterested for a whole week, n (%) | 98841  (22.03) | 74010  (21.99) | 24831  (22.13) | 1 |
| Often feels fed-up, n (%) | 166944  (37.20) | 125251  (37.22) | 41693  (37.16) | 1 |
| Felt depressed on several days over the past two weeks, n (%) | 72781  (16.22) | 54556  (16.21) | 18225  (16.25) | 1 |
| Has been depressed nearly every day over the past two weeks, n (%) | 5347  (1.19) | 4022  (1.20) | 1325  (1.18) | 1 |
| Has not felt tired in the last 2 weeks, n (%) | 211783  (47.20) | 158854  (47.20) | 52929  (47.18) | 1 |
| Experienced serious illness, injury or assault to themselves in the last 2 years, n (%) | 39655  (8.84) | 29750  (8.84) | 9905  (8.83) | 1 |
| Experienced financial difficulties in the past 2 years, n (%) | 50564  (11.27) | 37970  (11.28) | 12594  (11.23) | 1 |
| Mother diagnosed with severe depression, n (%) | 24311  (5.42) | 18299  (5.44) | 6012  (5.36) | 1 |
| Sometimes feels miserable for no reason, n (%) | 175732  (39.16) | 131851  (39.18) | 43881  (39.12) | 1 |
| Experiences mood swings, n (%) | 186162  (41.49) | 139718  (41.51) | 46444  (41.40) | 1 |
| More a 'morning' than 'evening' person, n (%) | 143841  (32.05) | 107871  (32.05) | 35970  (32.06) | 1 |
| Number of operations, median [Q1,Q3] | 1.00  [1.00,2.00] | 1.00  [1.00,2.00] | 1.00  [1.00,2.00] | 1 |
| Fair self-rated overall health, n (%) | 89385  (19.92) | 66899  (19.88) | 22486  (20.04) | 1 |
| Poor self-rated overall health, n (%) | 16305  (3.63) | 12253  (3.64) | 4052  (3.61) | 1 |
| Tried smoking just once or twice in the past, n (%) | 66197  (14.75) | 49465  (14.70) | 16732  (14.91) | 1 |
| Never smoked, n (%) | 181289  (40.40) | 136123  (40.45) | 45166  (40.26) | 1 |
| Does not have university degree, A/O levels, GCSE/CSE or professional qualifications, n (%) | 74751  (16.66) | 56112  (16.67) | 18639  (16.61) | 1 |
| CSEs or equivalent qualification, n (%) | 57973  (12.92) | 43479  (12.92) | 14494  (12.92) | 1 |
| Prefers not to say about seeing a psychiatrist for nerves, anxiety, tension or depression, n (%) | 902  (0.20) | 680  (0.20) | 222  (0.20) | 1 |
| Has seen a psychiatrist for nerves, anxiety, tension or depression, n (%) | 36892  (8.22) | 27777  (8.25) | 9115  (8.13) | 1 |
| Has seen a GP for nerves, anxiety, tension or depression, n (%) | 127851  (28.49) | 95930  (28.50) | 31921  (28.45) | 1 |
| Their feelings are easily hurt, n (%) | 233900  (52.12) | 175354  (52.10) | 58546  (52.19) | 1 |
| Manic/irritable episodes ended up in needing treatment or caused problems in life, n (%) | 5589  (1.25) | 4191  (1.25) | 1398  (1.25) | 1 |
| Previous smoker, n (%) | 155178  (34.58) | 116421  (34.59) | 38757  (34.55) | 1 |
| Height, median [Q1,Q3] | 168.00 [162.00,175.00] | 168.00 [162.00,175.00] | 168.00 [162.00,175.00] | 1 |
| Suffers from nerves, n (%) | 80536  (17.95) | 60281  (17.91) | 20255  (18.06) | 1 |
| Not tense or highly strung, n (%) | 364868  (81.31) | 273568  (81.29) | 91300  (81.38) | 1 |
| Water intake, median [Q1,Q3] | 2.00  [1.00,4.00] | 2.00  [1.00,4.00] | 2.00  [1.00,4.00] | 1 |
| A worrier, n (%) | 238369  (53.12) | 178769  (53.12) | 59600  (53.13) | 1 |

**Supplementary Table 8: Comparison of train+validation and test anxiety datasets.** Final set of variables after feature selection is shown. Last column shows p-value after comparing the incident depression group with the non-depression group. Comparisons were performed using the Chi-squared test for categories and Kruskal-Wallis test for continuous variables.

|  | n (%) | | | P-Value (adjusted) |
| --- | --- | --- | --- | --- |
|  | Full dataset | Train + validation | Test |  |
| n | 477100 | 357825 | 119275 |  |
| Incident depression, n (%) | 22436  (4.70) | 16881  (4.72) | 5555  (4.66) | 1 |
| Time-to-event (outcome/censoring), median [Q1,Q3] | 208565  (43.72) | 156312  (43.68) | 52253  (43.81) | 1 |
| Annual household income over £100,000, n (%) | 22436  (4.70) | 16881  (4.72) | 5555  (4.66) | 1 |
| Experiences mood swings, n (%) | 208565  (43.72) | 156312  (43.68) | 52253  (43.81) | 1 |
| Number of operations, median [Q1,Q3] | 1.00  [1.00,3.00] | 1.00  [1.00,3.00] | 1.00  [1.00,2.00] | 1 |
| Fair self-rated overall health, n (%) | 98509  (20.65) | 73918  (20.66) | 24591  (20.62) | 1 |
| Poor self-rated overall health, n (%) | 20215  (4.24) | 15012  (4.20) | 5203  (4.36) | 0.664 |
| Their feelings are easily hurt, n (%) | 254762  (53.40) | 191139  (53.42) | 63623  (53.34) | 1 |
| Suffers from nerves, n (%) | 93905  (19.68) | 70606  (19.73) | 23299  (19.53) | 1 |
| Water intake, median [Q1,Q3] | 2.00  [1.00,4.00] | 2.00  [1.00,4.00] | 2.00  [1.00,4.00] | 1 |
| Prefers not to answer about ability to confide, n (%) | 2727  (0.57) | 2012  (0.56) | 715  (0.60) | 1 |
| Age, median [Q1,Q3] | 58.00 [50.00,63.00] | 58.00 [50.00,63.00] | 58.00 [50.00,63.00] | 1 |
| Annual household income between £31,000 and £51,999, n (%) | 107393  (22.51) | 80346  (22.45) | 27047  (22.68) | 1 |
| Annual household income between £52,000 and £100,000, n (%) | 83995  (17.61) | 62716  (17.53) | 21279  (17.84) | 0.689 |
| Eats beef 2-4 times a week, n (%) | 53749  (11.27) | 40333  (11.27) | 13416  (11.25) | 1 |
| Body mass index, median [Q1,Q3] | 26.73 [24.13,29.88] | 26.73 [24.13,29.89] | 26.74 [24.14,29.88] | 1 |
| Smokes tobacco on most or all days, n (%) | 36519  (7.65) | 27365  (7.65) | 9154  (7.67) | 1 |
| Has been highly irritable or argumentative for two days in the past, n (%) | 130402  (27.33) | 97750  (27.32) | 32652  (27.38) | 1 |
| Has been unenthusiastic or disinterested for a whole week, n (%) | 55949  (11.73) | 41920  (11.72) | 14029  (11.76) | 1 |
| Has fractured bones in the last 5 years, n (%) | 44780  (9.39) | 33653  (9.40) | 11127  (9.33) | 1 |
| Has not felt depressed at all over the past two weeks, n (%) | 350632  (73.49) | 263101  (73.53) | 87531  (73.39) | 1 |
| Felt tense or restless nearly every day in the last 2 weeks, n (%) | 8154  (1.71) | 6194  (1.73) | 1960  (1.64) | 1 |
| Finds getting up in the morning fairly easy, n (%) | 237355  (49.75) | 178182  (49.80) | 59173  (49.61) | 1 |
| Finds getting up in the morning very easy, n (%) | 154696  (32.42) | 115892  (32.39) | 38804  (32.53) | 1 |
| Does not know the illnesses of father, n (%) | 32299  (6.77) | 24283  (6.79) | 8016  (6.72) | 1 |
| Prefers not to answer about the illnesses of father, n (%) | 546  (0.11) | 409  (0.11) | 137  (0.11) | 1 |
| Sibling not diagnosed with any of the diseases, n (%) | 252024  (52.82) | 189130  (52.86) | 62894  (52.73) | 1 |
| Sibling diagnosed with severe depression, n (%) | 26437  (5.54) | 19904  (5.56) | 6533  (5.48) | 1 |
| Not an irritable person, n (%) | 328959  (68.95) | 246845  (68.98) | 82114  (68.84) | 1 |
| Job never or rarely involves heavy manual/physical work, n (%) | 179228  (37.57) | 134344  (37.54) | 44884  (37.63) | 1 |
| Job sometimes involves heavy manual/physical work, n (%) | 58968  (12.36) | 44263  (12.37) | 14705  (12.33) | 1 |
| Pain in leg when walking, n (%) | 36738  (7.70) | 27586  (7.71) | 9152  (7.67) | 1 |
| Longest manic/irritable episode lasted 2-7 days, n (%) | 16141  (3.38) | 12080  (3.38) | 4061  (3.40) | 1 |
| Never feels miserable for no reason, n (%) | 270522  (56.70) | 202808  (56.68) | 67714  (56.77) | 1 |
| Never/rarely takes a nap during the day, n (%) | 268996  (56.38) | 201990  (56.45) | 67006  (56.18) | 1 |
| Not a nervous person, n (%) | 357344  (74.90) | 267872  (74.86) | 89472  (75.01) | 1 |
| Excellent self-rated overall health, n (%) | 79290  (16.62) | 59609  (16.66) | 19681  (16.50) | 1 |
| Headache experienced in the last month, n (%) | 96776  (20.28) | 72612  (20.29) | 24164  (20.26) | 1 |
| Stomach or abdominal pain experienced in the last month, n (%) | 41006  (8.59) | 30982  (8.66) | 10024  (8.40) | 0.332 |
| Pain all over the body experienced in the last month, n (%) | 7958  (1.67) | 5875  (1.64) | 2083  (1.75) | 0.744 |
| College or university degree, n (%) | 155742  (32.64) | 116878  (32.66) | 38864  (32.58) | 1 |
| Has not seen a psychiatrist for nerves, anxiety, tension or depression, n (%) | 423348  (88.73) | 317624  (88.77) | 105724  (88.64) | 1 |
| Has not seen a GP for nerves, anxiety, tension or depression, n (%) | 317871  (66.63) | 238399  (66.62) | 79472  (66.63) | 1 |
| Sometimes has trouble falling asleep or wakes up at night, n (%) | 227456  (47.67) | 170395  (47.62) | 57061  (47.84) | 1 |
| Usually has trouble falling asleep or wakes up at night, n (%) | 133409  (27.96) | 100261  (28.02) | 33148  (27.79) | 1 |
| Close partner or relative complains about their snoring, n (%) | 165366  (34.66) | 123886  (34.62) | 41480  (34.78) | 1 |
| Tense or highly strung, n (%) | 78889  (16.54) | 59143  (16.53) | 19746  (16.56) | 1 |
| Not a worrier, n (%) | 204146  (42.79) | 152909  (42.73) | 51237  (42.96) | 1 |

**Supplementary Table 9: Summary of demographic characteristics of the studied cohort grouped by the outcomes for depression.** Final set of variables after feature selection is shown. Last column shows p-value after comparing the incident depression group with the non-depression group. Comparisons were performed using the Chi-squared test for categories and Kruskal-Wallis test for continuous variables.

|  | n (%) | | | P-Value (adjusted) |
| --- | --- | --- | --- | --- |
|  | Overall | No depression | Depression |  |
| n | 336549 | 324169 | 12380 |  |
| A worrier, n (%) | 178769  (53.12) | 170048  (52.46) | 8721  (70.44) | <0.001 |
| Able to confide almost daily, n (%) | 176910  (52.57) | 171384  (52.87) | 5526  (44.64) | <0.001 |
| Annual household income less than ¬£18,000, n (%) | 61766  (18.35) | 58274  (17.98) | 3492  (28.21) | <0.001 |
| Annual household income over ¬£100,000, n (%) | 16248  (4.83) | 16004  (4.94) | 244  (1.97) | <0.001 |
| CSEs or equivalent qualification, n (%) | 43479  (12.92) | 41577  (12.83) | 1902  (15.36) | <0.001 |
| Does not have university degree, A/O levels, GCSE/CSE or professional qualifications, n (%) | 56112  (16.67) | 53305  (16.44) | 2807  (22.67) | <0.001 |
| Experienced financial difficulties in the past 2 years, n (%) | 37970  (11.28) | 35316  (10.89) | 2654  (21.44) | <0.001 |
| Experienced serious illness, injury or assault to themselves in the last 2 years, n (%) | 29750  (8.84) | 27803  (8.58) | 1947  (15.73) | <0.001 |
| Experiences mood swings, n (%) | 139718  (41.51) | 131292  (40.50) | 8426  (68.06) | <0.001 |
| Fair self-rated overall health, n (%) | 66899  (19.88) | 62900  (19.40) | 3999  (32.30) | <0.001 |
| Felt depressed on several days over the past two weeks, n (%) | 54556  (16.21) | 50663  (15.63) | 3893  (31.45) | <0.001 |
| Has been depressed nearly every day over the past two weeks, n (%) | 4022  (1.20) | 3375  (1.04) | 647  (5.23) | <0.001 |
| Has been depressed or down for a whole week in the past, n (%) | 56146  (16.68) | 53155  (16.40) | 2991  (24.16) | <0.001 |
| Has not been unenthusiastic or disinterested for a whole week, n (%) | 74010  (21.99) | 72598  (22.40) | 1412  (11.41) | <0.001 |
| Has not felt tired in the last 2 weeks, n (%) | 158854  (47.20) | 155560  (47.99) | 3294  (26.61) | <0.001 |
| Has seen a GP for nerves, anxiety, tension or depression, n (%) | 95930  (28.50) | 87703  (27.05) | 8227  (66.45) | <0.001 |
| Has seen a psychiatrist for nerves, anxiety, tension or depression, n (%) | 27777  (8.25) | 24528  (7.57) | 3249  (26.24) | <0.001 |
| Height, median [Q1,Q3] | 168.00 [162.00,175.00] | 168.00 [162.00,175.00] | 166.00 [160.00,173.30] | <0.001 |
| Manic/irritable episodes ended up in needing treatment or caused problems in life, n (%) | 4191  (1.25) | 3700  (1.14) | 491  (3.97) | <0.001 |
| More a 'morning' than 'evening' person, n (%) | 107871  (32.05) | 104243  (32.16) | 3628  (29.31) | <0.001 |
| Mother diagnosed with severe depression, n (%) | 18299  (5.44) | 17170  (5.30) | 1129  (9.12) | <0.001 |
| Never drinks alcohol, n (%) | 25079  (7.45) | 23652  (7.30) | 1427  (11.53) | <0.001 |
| Never smoked, n (%) | 136123  (40.45) | 131873  (40.68) | 4250  (34.33) | <0.001 |
| Not tense or highly strung, n (%) | 273568  (81.29) | 265649  (81.95) | 7919  (63.97) | <0.001 |
| Number of operations, median [Q1,Q3] | 1.00  [1.00,2.00] | 1.00  [1.00,2.00] | 2.00  [1.00,3.00] | <0.001 |
| Often feels fed-up, n (%) | 125251  (37.22) | 117560  (36.27) | 7691  (62.12) | <0.001 |
| Poor self-rated overall health, n (%) | 12253  (3.64) | 10762  (3.32) | 1491  (12.04) | <0.001 |
| Prefers not to say about seeing a psychiatrist for nerves, anxiety, tension or depression, n (%) | 680  (0.20) | 614  (0.19) | 66  (0.53) | <0.001 |
| Previous smoker, n (%) | 116421  (34.59) | 111956  (34.54) | 4465  (36.07) | 0.017 |
| Sometimes feels miserable for no reason, n (%) | 131851  (39.18) | 123917  (38.23) | 7934  (64.09) | <0.001 |
| Suffers from nerves, n (%) | 60281  (17.91) | 56109  (17.31) | 4172  (33.70) | <0.001 |
| Their feelings are easily hurt, n (%) | 175354  (52.10) | 166939  (51.50) | 8415  (67.97) | <0.001 |
| Tried smoking just once or twice in the past, n (%) | 49465  (14.70) | 47964  (14.80) | 1501  (12.12) | <0.001 |
| Unable to work because of sickness or disability, n (%) | 10006  (2.97) | 8750  (2.70) | 1256  (10.15) | <0.001 |
| Water intake, median [Q1,Q3] | 2.00  [1.00,4.00] | 2.00  [1.00,4.00] | 2.00  [1.00,4.00] | <0.001 |

**Supplementary Table 10: Summary of demographic characteristics of the studied cohort grouped by the outcomes for anxiety.** Final set of variables after feature selection is shown. Last column shows p-value after comparing the incident anxiety group with the non-anxiety group. Comparisons were performed using the Chi-squared test for categories and Kruskal-Wallis test for continuous variables.

|  | n (%) | | | P-Value (adjusted) |
| --- | --- | --- | --- | --- |
|  | Overall | No anxiety | Anxiety |  |
| n | 357825 | 344453 | 13372 |  |
| Age, median [Q1,Q3] | 58.00 [50.00,63.00] | 58.00 [50.00,63.00] | 58.00 [50.00,64.00] | 0.405 |
| Annual household income between £31,000 and £51,999, n (%) | 80346  (22.45) | 77892  (22.61) | 2454  (18.35) | <0.001 |
| Annual household income between £52,000 and £100,000, n (%) | 62716  (17.53) | 61214  (17.77) | 1502  (11.23) | <0.001 |
| Annual household income over £100,000, n (%) | 16881  (4.72) | 16646  (4.83) | 235  (1.76) | <0.001 |
| Body mass index, median [Q1,Q3] | 26.73 [24.13,29.89] | 26.71 [24.13,29.86] | 27.11 [24.26,30.80] | <0.001 |
| Close partner or relative complains about their snoring, n (%) | 123886 (34.62) | 119468 (34.68) | 4418  (33.04) | 0.004 |
| College or university degree, n (%) | 116878 (32.66) | 113527 (32.96) | 3351  (25.06) | <0.001 |
| Does not know the illnesses of father, n (%) | 24283  (6.79) | 23255  (6.75) | 1028  (7.69) | 0.001 |
| Eats beef 2-4 times a week, n (%) | 40333  (11.27) | 39088  (11.35) | 1245  (9.31) | <0.001 |
| Excellent self-rated overall health, n (%) | 59609  (16.66) | 58613  (17.02) | 996  (7.45) | <0.001 |
| Experiences mood swings, n (%) | 156312 (43.68) | 147381 (42.79) | 8931  (66.79) | <0.001 |
| Fair self-rated overall health, n (%) | 73918  (20.66) | 69654  (20.22) | 4264  (31.89) | <0.001 |
| Felt tense or restless nearly every day in the last 2 weeks, n (%) | 6194  (1.73) | 5398  (1.57) | 796  (5.95) | <0.001 |
| Finds getting up in the morning fairly easy, n (%) | 178182 (49.80) | 171979 (49.93) | 6203  (46.39) | <0.001 |
| Finds getting up in the morning very easy, n (%) | 115892 (32.39) | 112585 (32.69) | 3307  (24.73) | <0.001 |
| Has been highly irritable or argumentative for two days in the past, n (%) | 97750  (27.32) | 94620  (27.47) | 3130  (23.41) | <0.001 |
| Has been unenthusiastic or disinterested for a whole week, n (%) | 41920  (11.72) | 39100  (11.35) | 2820  (21.09) | <0.001 |
| Has fractured bones in the last 5 years, n (%) | 33653  (9.40) | 32042  (9.30) | 1611  (12.05) | <0.001 |
| Has not felt depressed at all over the past two weeks, n (%) | 263101 (73.53) | 256282 (74.40) | 6819  (50.99) | <0.001 |
| Has not seen a GP for nerves, anxiety, tension or depression, n (%) | 238399 (66.62) | 234457 (68.07) | 3942  (29.48) | <0.001 |
| Has not seen a psychiatrist for nerves, anxiety, tension or depression, n (%) | 317624 (88.77) | 308585 (89.59) | 9039  (67.60) | <0.001 |
| Headache experienced in the last month, n (%) | 72612  (20.29) | 68666  (19.93) | 3946  (29.51) | <0.001 |
| Job never or rarely involves heavy manual/physical work, n (%) | 134344 (37.54) | 130485 (37.88) | 3859  (28.86) | <0.001 |
| Job sometimes involves heavy manual/physical work, n (%) | 44263  (12.37) | 42827  (12.43) | 1436  (10.74) | <0.001 |
| Longest manic/irritable episode lasted 2-7 days, n (%) | 12080  (3.38) | 11392  (3.31) | 688  (5.15) | <0.001 |
| Never feels miserable for no reason, n (%) | 202808 (56.68) | 198222 (57.55) | 4586  (34.30) | <0.001 |
| Never/rarely takes a nap during the day, n (%) | 201990 (56.45) | 195469 (56.75) | 6521  (48.77) | <0.001 |
| Not a nervous person, n (%) | 267872 (74.86) | 260861 (75.73) | 7011  (52.43) | <0.001 |
| Not a worrier, n (%) | 152909 (42.73) | 150066 (43.57) | 2843  (21.26) | <0.001 |
| Not an irritable person, n (%) | 246845 (68.98) | 239144 (69.43) | 7701  (57.59) | <0.001 |
| Number of operations, median [Q1,Q3] | 1.00 [1.00,3.00] | 1.00 [1.00,2.00] | 2.00 [1.00,3.00] | <0.001 |
| Pain all over the body experienced in the last month, n (%) | 5875  (1.64) | 5331  (1.55) | 544  (4.07) | <0.001 |
| Pain in leg when walking, n (%) | 27586  (7.71) | 25935  (7.53) | 1651  (12.35) | <0.001 |
| Poor self-rated overall health, n (%) | 15012  (4.20) | 13445  (3.90) | 1567  (11.72) | <0.001 |
| Prefers not to answer about ability to confide, n (%) | 2012  (0.56) | 1910  (0.55) | 102  (0.76) | 0.092 |
| Prefers not to answer about the illnesses of father, n (%) | 409  (0.11) | 380  (0.11) | 29  (0.22) | 0.027 |
| Sibling diagnosed with severe depression, n (%) | 19904  (5.56) | 18527  (5.38) | 1377  (10.30) | <0.001 |
| Sibling not diagnosed with any of the diseases, n (%) | 189130 (52.86) | 182945 (53.11) | 6185  (46.25) | <0.001 |
| Smokes tobacco on most or all days, n (%) | 27365  (7.65) | 25811  (7.49) | 1554  (11.62) | <0.001 |
| Sometimes has trouble falling asleep or wakes up at night, n (%) | 170395 (47.62) | 164376 (47.72) | 6019  (45.01) | <0.001 |
| Stomach or abdominal pain experienced in the last month, n (%) | 30982  (8.66) | 28876  (8.38) | 2106  (15.75) | <0.001 |
| Suffers from nerves, n (%) | 70606  (19.73) | 64941  (18.85) | 5665  (42.36) | <0.001 |
| Tense or highly strung, n (%) | 59143  (16.53) | 54324  (15.77) | 4819  (36.04) | <0.001 |
| Their feelings are easily hurt, n (%) | 191139 (53.42) | 181660 (52.74) | 9479  (70.89) | <0.001 |
| Usually has trouble falling asleep or wakes up at night, n (%) | 100261 (28.02) | 94839  (27.53) | 5422  (40.55) | <0.001 |
| Water intake, median [Q1,Q3] | 2.00 [1.00,4.00] | 2.00 [1.00,4.00] | 2.00 [1.00,4.00] | <0.001 |

**Supplementary Table 11: Summary of the Cox Proportional Hazards model for manually excluded variables.** The table displays coefficients = log(HR) with 95% confidence intervals and -log2(p-value). All columns were statistically significant (where p < 0.05 and null hypothesis states that the coefficient is equal to 0).

| **Covariate** | **log(HR)** | **CI log(HR) lower 95%** | **CI log(HR) upper 95%** | **-log2**  **(p-value)** |
| --- | --- | --- | --- | --- |
| **Depression** |  |  |  |  |
| Very unhappy with their financial situation | -0.0679592 | -0.1950968 | 0.05917839 | 1.76222551 |
| Diagnosed with other serious medical condition | -0.1155732 | -0.1574639 | -0.0736826 | 23.898513 |
| Does not worry too long after embarrassment | 0.11995798 | 0.07915548 | 0.16076048 | 26.8440592 |
| Felt tired on several days in the last 2 weeks | -0.1599073 | -0.2047945 | -0.11502 | 38.3243001 |
| **Anxiety** |  |  |  |  |
| Prefers not to answer about feeling tense or restless in the last 2 weeks | -0.0286258 | -0.296215 | 0.2389634 | 0.26201042 |
| Prefers not to answer about pain experienced in the last month | -0.0569121 | -0.38498 | 0.27115584 | 0.44644137 |
| Diagnosed with other serious medical condition | -0.0683524 | -0.1082363 | -0.0284685 | 10.3198313 |
| Does not worry too long after embarrassment | 0.08429794 | 0.04388342 | 0.12471246 | 14.4892464 |
| Their feelings are not easily hurt | 0.03499848 | -0.0732273 | 0.14322428 | 0.92632296 |
| Sibling diagnosed with high blood pressure | -0.0896193 | -0.1384311 | -0.0408075 | 11.6095329 |
| **Never or almost never able to confide** | **-0.0318744** | **-0.0783528** | **0.01460404** | **2.48270102** |

**Supplementary Table 12: Summary of the Cox Proportional Hazards model for depression.** The table displays coefficients = log(HR) with 95% confidence intervals and -log2(p-value). All columns were statistically significant (where p < 0.05 and null hypothesis states that the coefficient is equal to 0).

| **Covariate** | **log(HR)** | **CI log(HR) lower 95%** | **CI log(HR) upper 95%** | **-log2**  **(p-value)** |
| --- | --- | --- | --- | --- |
| Has seen a GP for nerves, anxiety, tension or depression | 1.038 | 0.995 | 1.082 | >1000 |
| Prefers not to say about seeing a psychiatrist for nerves, anxiety, tension or depression | 0.933 | 0.691 | 1.176 | 44.271 |
| Poor self-rated overall health | 0.513 | 0.445 | 0.581 | 163.422 |
| Has been depressed nearly every day over the past two weeks | 0.413 | 0.327 | 0.500 | 66.788 |
| Has seen a psychiatrist for nerves, anxiety, tension or depression | 0.340 | 0.295 | 0.385 | 162.836 |
| Fair self-rated overall health | 0.317 | 0.275 | 0.359 | 163.503 |
| Experiences mood swings | 0.290 | 0.242 | 0.338 | 105.019 |
| Unable to work because of sickness or disability | 0.228 | 0.161 | 0.296 | 34.724 |
| Experienced serious illness, injury or assault to themselves in the last 2 years | 0.202 | 0.151 | 0.254 | 46.181 |
| Manic/irritable episodes ended up in needing treatment or caused problems in life | 0.186 | 0.089 | 0.283 | 12.572 |
| Never drinks alcohol | 0.182 | 0.125 | 0.239 | 31.378 |
| Often feels fed-up | 0.179 | 0.133 | 0.225 | 45.157 |
| Does not have university degree, A/O levels, GCSE/CSE or professional qualifications | 0.168 | 0.122 | 0.214 | 40.561 |
| Sometimes feels miserable for no reason | 0.162 | 0.116 | 0.208 | 37.930 |
| Felt depressed on several days over the past two weeks | 0.161 | 0.118 | 0.204 | 41.964 |
| Annual household income less than £18,000 | 0.150 | 0.108 | 0.192 | 38.240 |
| Experienced financial difficulties in the past 2 years | 0.146 | 0.100 | 0.192 | 31.250 |
| Has been depressed or down for a whole week in the past | 0.131 | 0.085 | 0.176 | 26.064 |
| CSEs or equivalent qualification | 0.127 | 0.076 | 0.178 | 20.085 |
| Mother diagnosed with severe depression | 0.117 | 0.055 | 0.178 | 12.164 |
| Suffers from nerves | 0.092 | 0.049 | 0.134 | 15.307 |
| Number of operations | 0.086 | 0.076 | 0.096 | 207.829 |
| A worrier | 0.054 | 0.010 | 0.099 | 5.958 |
| Their feelings are easily hurt | 0.049 | 0.007 | 0.091 | 5.434 |
| Water intake | 0.027 | 0.020 | 0.034 | 42.864 |
| Height | -0.003 | -0.005 | -0.001 | 9.542 |
| More a 'morning' than 'evening' person | -0.016 | -0.055 | 0.023 | 1.244 |
| Able to confide almost daily | -0.039 | -0.075 | -0.003 | 4.806 |
| Not tense or highly strung | -0.115 | -0.157 | -0.072 | 23.010 |
| Has not felt tired in the last 2 weeks | -0.190 | -0.235 | -0.146 | 54.312 |
| Previous smoker | -0.238 | -0.290 | -0.185 | 60.578 |
| Has not been unenthusiastic or disinterested for a whole week | -0.243 | -0.301 | -0.186 | 52.981 |
| Never smoked | -0.327 | -0.380 | -0.273 | 107.738 |
| Tried smoking just once or twice in the past | -0.343 | -0.411 | -0.276 | 75.893 |
| Annual household income over £100,000 | -0.463 | -0.590 | -0.336 | 39.834 |


**Supplementary Table 13: Summary of the Cox Proportional Hazards model for anxiety.** The table displays coefficients = log(HR) with 95% confidence intervals and -log2(p-value). All columns were statistically significant (where p < 0.05 and null hypothesis states that the coefficient is equal to 0).


| **Covariate** | **log(HR)** | **CI log(HR) lower 95%** | **CI log(HR) upper 95%** | **-log2**  **(p-value)** |
| --- | --- | --- | --- | --- |
| Prefers not to answer about the illnesses of father | 0.482 | 0.133 | 0.832 | 7.205 |
| Poor self-rated overall health | 0.304 | 0.237 | 0.370 | 61.546 |
| Suffers from nerves | 0.262 | 0.219 | 0.305 | 107.323 |
| Has been unenthusiastic or disinterested for a whole week | 0.220 | 0.167 | 0.273 | 51.031 |
| Pain all over the body experienced in the last month | 0.206 | 0.114 | 0.297 | 16.546 |
| Fair self-rated overall health | 0.199 | 0.158 | 0.241 | 67.644 |
| Sibling diagnosed with severe depression | 0.195 | 0.138 | 0.252 | 35.715 |
| Tense or highly strung | 0.183 | 0.140 | 0.226 | 53.162 |
| Smokes tobacco on most or all days | 0.156 | 0.101 | 0.211 | 25.319 |
| Experiences mood swings | 0.139 | 0.094 | 0.185 | 28.566 |
| Stomach or abdominal pain experienced in the last month | 0.133 | 0.083 | 0.182 | 22.527 |
| Usually has trouble falling asleep or wakes up at night | 0.130 | 0.076 | 0.185 | 18.437 |
| Felt tense or restless nearly every day in the last 2 weeks | 0.128 | 0.051 | 0.206 | 9.787 |
| Prefers not to answer about ability to confide | 0.125 | -0.066 | 0.315 | 2.323 |
| Not an irritable person | 0.102 | 0.063 | 0.141 | 22.102 |
| Has fractured bones in the last 5 years | 0.100 | 0.048 | 0.153 | 12.512 |
| Sometimes has trouble falling asleep or wakes up at night | 0.095 | 0.044 | 0.147 | 11.682 |
| Their feelings are easily hurt | 0.087 | 0.046 | 0.128 | 14.779 |
| Pain in leg when walking | 0.085 | 0.025 | 0.145 | 7.455 |
| Number of operations | 0.076 | 0.061 | 0.092 | 70.702 |
| Headache experienced in the last month | 0.072 | 0.032 | 0.112 | 11.13 |
| Does not know the illnesses of father | 0.052 | -0.011 | 0.116 | 3.211 |
| Has been highly irritable or argumentative for two days in the past | 0.043 | -0.005 | 0.091 | 3.679 |
| Age | 0.038 | 0.017 | 0.059 | 11.171 |
| Water intake | 0.029 | 0.013 | 0.045 | 10.937 |
| Longest manic/irritable episode lasted 2-7 days | 0.017 | -0.066 | 0.101 | 0.551 |
| Body mass index | 0.007 | -0.010 | 0.024 | 1.287 |
| Never/rarely takes a nap during the day | -0.050 | -0.085 | -0.015 | 7.683 |
| Sibling not diagnosed with any of the diseases | -0.053 | -0.088 | -0.018 | 8.398 |
| Finds getting up in the morning very easy | -0.068 | -0.118 | -0.018 | 6.959 |
| Finds getting up in the morning fairly easy | -0.069 | -0.112 | -0.026 | 9.334 |
| Close partner or relative complains about their snoring | -0.072 | -0.109 | -0.035 | 12.881 |
| Job never or rarely involves heavy manual/physical work | -0.098 | -0.143 | -0.053 | 15.654 |
| Job sometimes involves heavy manual/physical work | -0.105 | -0.164 | -0.047 | 11.128 |
| College or university degree | -0.106 | -0.148 | -0.064 | 20.563 |
| Never feels miserable for no reason | -0.110 | -0.154 | -0.066 | 19.709 |
| Has not felt depressed at all over the past two weeks | -0.131 | -0.172 | -0.090 | 30.693 |
| Annual household income between £31,000 and £51,999 | -0.145 | -0.192 | -0.098 | 28.95 |
| Eats beef 2-4 times a week | -0.168 | -0.226 | -0.110 | 26.507 |
| Not a nervous person | -0.207 | -0.250 | -0.163 | 66.674 |
| Annual household income between £52,000 and £100,000 | -0.21 | -0.269 | -0.150 | 37.07 |
| Not a worrier | -0.266 | -0.314 | -0.218 | 88.85 |
| Excellent self-rated overall health | -0.281 | -0.348 | -0.214 | 51.909 |
| Has not seen a psychiatrist for nerves, anxiety, tension or depression | -0.337 | -0.379 | -0.296 | 187.872 |
| Annual household income over £100,000 | -0.531 | -0.662 | -0.400 | 49.000 |
| Has not seen a GP for nerves, anxiety, tension or depression | -0.94 | -0.984 | -0.897 | > 1000 |
